# RNA-Binding Protein COL14A1, TNS1, NUSAP1 and YWHAE Are Valid Biomarkers to Predict Peritoneal Metastasis in Gastric Cancer

**DOI:** 10.3389/fonc.2022.830688

**Published:** 2022-04-19

**Authors:** Yue Jiang, Fangfang Chen, Xunshan Ren, Yu Yang, Jiajun Luo, Jingwen Yuan, Jingping Yuan, Qiang Tong

**Affiliations:** ^1^ Department of Gastrointestinal Surgery I Section, Renmin Hospital of Wuhan University, Wuhan, China; ^2^ Department of Pathology, Renmin Hospital of Wuhan University, Wuhan, China; ^3^ Department of Orthopedics, Renmin Hospital of Wuhan University, Wuhan, China

**Keywords:** gastric cancer, RNA-binding protein, peritoneal metastasis, prediction model, survival analysis

## Abstract

Gastric cancer (GC) is the third leading cause of tumor related mortality worldwide. Peritoneal metastasis (PM) occurs in more than half of advanced GC patients, leading to poor prognosis. Therefore, the GSE62254 cohort was used to construct a signature consisting of four RNA-binding proteins (RBP) to predict the possibility of PM in GC patients. Then, ROC curves were plotted followed by calculation of AUCs, showing that the signature had a similar predictive accuracy compared with the TNM staging system. Importantly, the capability of prediction was enhanced by combining the classifier and TNM staging. In order to validate the expression of the four RBPs in GC tissues with and without PM, immunohistochemistry was further performed on samples from 108 patients. The differential expression of COL14A1, TNS1, NUSAP1 and YWHAE was in accordance with the emergence of PM. Afterwards, we produced Kaplan–Meier curves according to the signature and differential expression of the RBPs in patients. Finally, CCK-8 assays were performed to verify the effect on cell proliferation, finding that COL14A1 and TNS1 promoted cell proliferation, while NUSAP1 and YWHAE led to suppressed cell proliferation. In conclusion, the four-RBP-based signature, combined with TNM staging, has the potential to predict risk of PM in GC.

## Introduction

Gastric cancer (GC) is one of the most common malignant tumors as well as the third leading cause of mortality all over the world (1). The incidence of GC ranks third among all malignant tumors in China, being next to lung and hepatic cancer (2). According to a global survey, there were over one million new GC cases in 2020, with 769000 estimated deaths (3). While surgery, radiotherapy, chemotherapy and biological treatment have been adopted heretofore, the 5-year overall survival rate of GC is still poor. Nearly 50% of GC patients have unspecific gastrointestinal symptoms and debilitating features are usually present at advanced stages in most cases (4). Peritoneal metastasis (PM) occurs in about 53–66% of advanced GC patients, leading to poor prognosis (5). Regrettably, effective treatments for peritoneal metastases are still lacking due to little understanding of the underlying mechanisms.

RNA-binding proteins (RBPs) are key players in post-transcriptional events which regulate the process of tumorigenesis, and each step leading to the initiation of malignancy may involve one or more RBPs (6). Mechanisms of RBPs regulation have been identified in cancer cells, including alternative splicing, polyadenylation, stability, subcellular localization, translation and so on (7). Several studies have provided immunohistochemical evidence that RBPs are abnormally expressed in cancers relative to adjacent normal tissues, and this expression correlates with patient prognosis (8–10). Besides, RBPs also interact with different coding or non-coding RNAs, such as microRNAs (miRNAs), long non-coding RNAs (lncRNAs) and circular RNAs (circRNAs) (11).

In consideration of the enormous influence of PM on the prognosis of GC patients, prediction of the risk has recently been a research focus. In clinical practice, miRNAs, lncRNAs, circRNAs and pathological factors including TNM staging and lymph node status have been gradually used to assess GC prognosis (12). Recently, studies have revealed that RBPs were associated with the prognosis of GC patients (13–15). Therefore, we proposed to recognize several RBPs as potential biomarkers based on transcriptome analysis to predict whether peritoneal metastasis would occur on GC patients. As a result, a 4-RBP-based classifier was constructed by use of Lasso Logistics, which could optimize the predictivity in combination with the current TNM staging system. Our results demonstrated that the 4-RBP-based classifier could be used as a reliable prognostic predictor of peritoneal metastasis in GC patients.

## Materials and Methods

### Data Acquisition

Transcriptome profiling data of tumor tissues in 300 GC samples with and without peritoneal metastasis were obtained from the GSE62254 cohort. For the purpose of analyzing the correlation between gene expression signatures and the occurrence of PM in GC patients, we filtered out 79 samples whose first sites of recurrence were not peritoneal seeding or ascites. Finally, 221 patients were selected and divided into training set (n = 147) and validating set (n = 74) randomly at a 2:1 ratio for further analysis. A total of 846 genes coding for RBPs were summarized from the published literature.

### Data Processing and Risk Score Calculation

737 RBPs examined in the GSE62254 cohort were subjected to Univariate Logistics analysis to select RBPs relevant to the occurrence of PM in GC patients. We selected the top 100 RBPs into Lasso Logistics analysis to acquire the coefficients. Then, four significantly correlated RBPs weighted by their coefficients were recognized to establish the prediction signature. After comparison and combination with TNM staging, a risk score formula for risk of PD in GC was constructed and demonstrated by a nomogram.

### Pathway Enrichment Analysis

DAVID (version 6.7) (https://david-d.ncifcrf.gov/), an online bioinformatics analysis tool was used to perform the gene-Gene Ontology (GO) term and the Kyoto Encyclopedia of Genes and Genomes (KEGG) enrichment analysis. Metascape (https://metascape.org/gp/index.html#/main/step1) offered complementary annotation.

### Patients and Tissue Samples

We went through the pathology database of Renmin Hospital of Wuhan University for GC patients with PM, finding 36 cases in the period from 2014 to 2021. Then we randomly chose 72 GC patients without PM in the year of 2016 as the control group. As a result, a total of 108 formalin-fixed, paraffin-embedded GC tissue samples were obtained. All the patients underwent surgical treatment at Renmin Hospital of Wuhan University and there were none previous chemotherapies, radiotherapies, or other treatments before surgery on these patients. The study was approved by the Ethics Committee of Renmin Hospital of Wuhan University.

### Immunohistochemical (IHC)

The paraffin tissues were cut into 4 um-thick sections, dried, dewaxed in xylene, and dehydrated in ascending series of ethanol. Antigen retrieval was conducted by microwave heating with citrate buffer (pH 6.0) for 20 min. Subsequently, paraffin sections were rinsed with PBS (3×5 min) and then blocked with 3% hydrogen peroxide at room temperature for endogenous peroxidase ablation for 25 min. Then the samples were exposed to Bovine Serum Albumin (BSA) at room temperature for 30 min to decrease nonspecific antibody binding after rinsing in PBS. The tissue sections were incubated overnight at 4°C with the primary antibody (anti-COL14A1, 1:200, ThermoFisher, America; anti-TNS1, 1:200, Abcam, British; anti-NUSAP1, 1:100, Abcam, British; anti-YWHAE, 1:500, Abcam, British);. After rinsing in PBS, the tissue sections were incubated with horseradish peroxidase-labeled anti-rabbit antibodies at room temperature for 30 min. Then, the tissue sections were rinsed with PBS for 4 times and then dripped with freshly prepared 3,3-diaminobenzidine (DAB). Microscopically, the staining was terminated when the tissue sections were brown-yellow or brown. Subsequently, all the tissue sections were restrained with hematoxylin for about 3 min. Finally, the slices were dehydrated with ethanol and toluene and then sealed with neutral gum. PBS was used to replace the primary antibody as a negative control. The slides were viewed *via* Olympus BX53 (Tokyo, Japan) microscope. IHC staining was evaluated independently by two pathologists under the double-blind condition. The staining intensity was classified as four grades as follows: 0 (no staining), 1 (light yellow), 2 (brown-yellow), and 3 (dark brown). The percentage of positive cells was classified as five grades as follows: 0 (0%), 1 (≤30%), 2 (31-50%), 3 (51-80%), and 4 (≥80%). Five most representative fields of high magnification (400x) were selected to calculate the final score. The final immunohistochemical score was the product of staining intensity and extent, theoretically from 0 to 12. Scores less than 4 were defined as low expression, and scores greater than or equal to 4 were described as high expression.

### Cell Lines

AGS cell line and MGC-803 cell line were bought from American Type Culture Collection (ATCC, Manassas, USA). AGS cell line was maintained in Dulbecco’s Modified Eagle Medium (DMEM)/F-12 (Servicebio, Wuhan, China) while MGC-803 cell line was maintained in DMEM-H (Servicebio, Wuhan, China), supplemented with 10% fetal bovine serum (ThermoFisher, America) and 1% antibiotics (Servicebio, Wuhan, China). Cells were maintained in a 37° C incubator with 5% CO2. All cell lines tested negative for mycoplasma.

### Cell Transfection

The siRNA vectors against COL14A1 or TNS1 were utilized for knockdown of COL14A1 or TNS1 with scrambled siRNA (siNC) as negative control. Sequences of the siRNAs were as follows: siCOL14A1-1: 5′‐GUGGUGGUAGAUGGAACUGUATT‐3′; siCOL14A1-2: 5′‐CUCAGGUUACCUGAUCCUUUATT‐3′; siTNS1-1: 5′‐CAGGUCUUACUCACCUUAUGATT‐3′; siTNS1-2: 5′‐GCAACUACCUGCUGUUCAATT‐3′. For upregulation of NUSAP1 or YWHAE, the full length of NUSAP1 or YWHAE was inserted into pcDNA3.1 vectors (Invitrogen) and the empty plasmids were served as negative control.

Cells were plated in 6-well plates with DMEM/F-12 medium supplemented with 10% medium FBS for 24 h before transfection. Transfections of siRNAs and indicated plasmids were both performed using Lipofectamine 2000 (ThermoFisher, America) according to the manufacturer’s instruction.

### Quantitative Real-Time PCR (qRT-PCR)

Total RNA from cells was extracted by TRIzol reagent (ThermoFisher, America) following the supplier’s instructions. Reverse transcription was conducted with the First Strand cDNA Synthesis Kit (Servicebio, Wuhan, China). PCR was implemented with SYBR Green qPCR Master Mix (Servicebio, Wuhan, China). The conditions for qRT-PCR were as follows: 95°C for 3 min, followed by 40 cycles of 10 s at 95°C, 10 s at 60°C, and 15 s at 70°C, followed by heating from 65°C to 95°C.

The sequences of main primers were as follows: COL14A1 (forward): 5′‐AGTGGGTGAGAAGGCAATGA‐3′, COL14A1 (reverse): 5′‐CTCTCAGGCCTGGAAGTTCA‐3′; TNS1 (forward):5’-TCAAGTGGAAGAACTTGTTTGCTT-3’, TNS1 (reverse): 5′‐CACGACAATATAGTGGAGGCACA‐3′; NUSAP1 (forward): 5′-AGCCCATCAATAAGGGAGGG-3′, NUSAP1 (reverse): 5′‐ACCTGACACCCGTTTTAGCTG‐3′; YWHAE (forward): 5′‐GCTGGATCCATGGATGATCGAGAGGATCTG‐3′, YWHAE (reverse): 5′‐GCTGAATTCTCACTGATTTTCGTCTTCCAC‐3′; GAPDH (forward): 5′‐CACCATTGGCAATGAGCGGTTC‐3′, GAPDH (reverse): 5′‐AGGTCTTTGCGGATGTCCACGT‐3′; GAPDH was utilized as an endogenous control.

### Cell Proliferation Assays

For Cell Counting Kit‐8 (CCK‐8) assay, transfected cells were inoculated at a density of 2 × 100 cells/well into 96‐well plates and cultivated for 0, 24, 48 and 72 hours. After different incubation times, each well was added with 10 μL of CCK‐8 reagent (Servicebio, Wuhan, China) and cultured for another hour. Then, the absorbance at 450 nm was recorded with a standard microplate reader (EnSight, Perkin Elmer, America).

### Statistical Analysis

We used Chi-squared test and Fisher’s exact test to measure both the difference between training and validating sets, and the difference between GC patients with and without PM. Univariable Logistics, Multivariate Logistics and Lasso Logistics analysis were performed using the R program. The Kaplan–Meier survival curves were drawn to demonstrate the relationship between the risk score and OS. The log-rank test was conducted to test the significance of all the Kaplan–Meier survival curves. ROC analysis was performed to measure prognostic accuracy. T-test was performed for statistical analyses in RT-qPCR and CCK-8 assay. All statistical tests were two-sided, and P < 0.05 was considered statistically significant. All analyses were performed in SPSS version 28.0.0 (SPSS Inc., Chicago, IL, United States) or R version 4.0.2 with the following packages: “heatmap”, “glmnet”, “gplot2” and “nsROC”.

## Results

### Data Source and Processing

**Figure 1:**
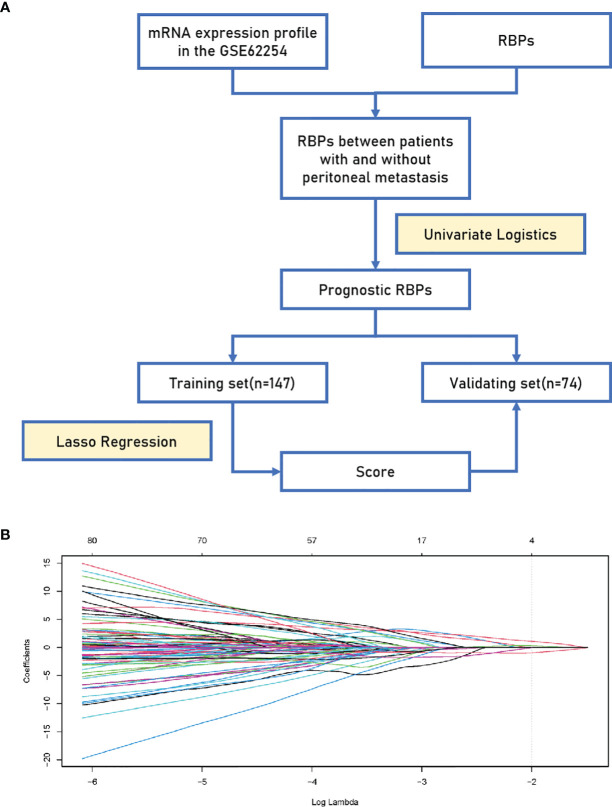
Establishment of the 4-RBP-based signature. **(A)** A flowchart showing the process of constructing the RBP-based signature to predict the possibility of peritoneal metastasis in gastric cancer. **(B)** LASSO coefficient profiles of the 100 gastric-cancer-associated RBPs. The vertical line represents the chosen Lambda value.

Originally, we obtained 300 GC samples from the GSE62254 cohort. Then, 79 samples whose first sites of recurrence were not peritoneal seeding or ascites were excluded. Afterwards, a list of 846 coding genes known or predicted as RBPs were matched with the 20174 genes examined in the GSE62254 cohort (16). Finally, 737 RBPs were subjected to a Univariate Logistics analysis and the top 100 RBPs ranked according to p-value were retained for further study. The clinical characters of GC patients were downloaded from the GSE62254 cohort. Then we divided the cases into a training set (n = 147) and a validating set (n = 74) at a 2:1 ratio randomly. No significant differences were seen between the two sets in gender, age, pathological stage, Lauren’s classification or lymphnode metastasis (**Table 1**). We eventually identified four RBPs strongly associated with the occurrence of PM in GC patients by Lasso Logistics analysis in the training set (**Figure 1**); chosen genes including Collagen Type XIV Alpha 1 Chain (COL14A1), Tensin 1 (TNS1), Nucleolar and Spindle Associated Protein 1 (NUSAP1) and Tyrosine 3-Monooxygenase/Tryptophan 5-Monooxygenase (YWHAE). Among the four genes, increased expression of COL14A1 and TNS1 was related to a higher risk of PM. Conversely, increased expression of NUSAP1 and YWHAE was associated with a lower risk of PM.

**Table 1:** Clinical features of GC patients in the training and validating sets.

Features	Training set (n=147)	Validating set (n=74)	Pearson χ2	P
**Gender**				
Male	96	44	0.725	0.395
Female	51	30		
**Age**				
<=65	64	33	0.022	0.881
>65	83	41		
**Pathological Stage**				
I+II	64	33	0.022	0.881
III+IV	83	41		
**Lymphnode Metastasis**				
Positive	92	54	1.627	0.202
Negative	42	16		
**Lauren’s Classification**				
Intestinal	65	37	0.662	0.416
Diffuse & Mixed	82	37		

### Establishment and Validation of a 4-RBP-Based Classifier to Predict the Risk for Peritoneal Metastasis in Gastric Cancer

**Figure 2:**
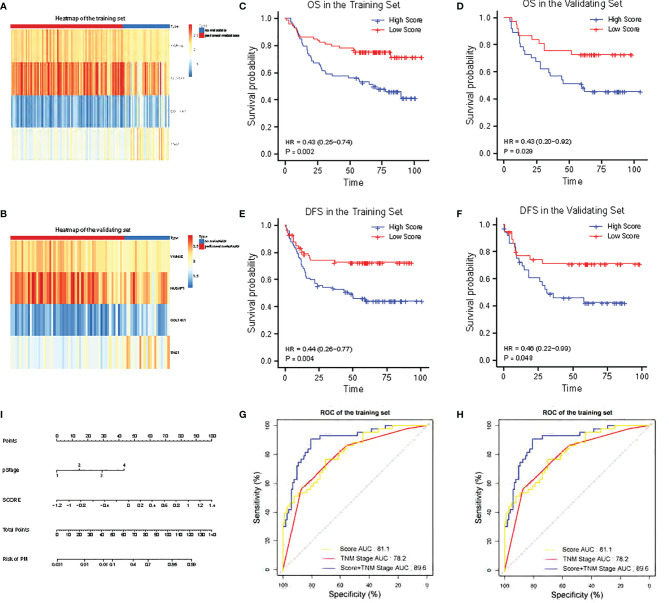
Validation of the 4-RBP-based signature in the training set and the validating set. **(A, B)** The heatmap of the four RBPs expression profiles. **(C, D)** Kaplan–Meier analysis for overall survival (OS) of GC patients based on the risk stratification. **(E, F)** Kaplan–Meier analysis for disease-free survival (DFS) of GC patients based on the risk stratification. **(G, H)** Receiver operating characteristic (ROC) analysis for the risk of PM including the risk score, TNM stage and combination of the two. **(I)** Nomogram to predict the risk of PM in GC.

The heatmaps revealed that COL14A1 and TNS1 were highly expressed in GC patients with PM in the training set, while NUSAP1 and YWHAE were highly expressed in the cases without PM (**Figure 2A**). Consistent results were observed in the validating set (**Figure 2B**). To assess the ability of the 4-RBP-based classifier to forecast the risk of PM in GC patients, we developed a risk score according to the coefficients of the four RBPs in Lasso Logistics: Risk Score = (1.08004578 * expression value of COL14A1) + (0.31114396 * expression value of TNS1) - (0.95036402 * expression value of NUSAP1) - (0.02147674 * expression value of YWHAE). The risk score formula was used to calculate the risk score in the training set, and the cases were divided into high-risk and low-risk groups owing to the cutoff of the median risk score. Kaplan–Meier curves showed that patients in the high-risk group had shorter overall survival than those in the low-risk group (p = 0.002) (**Figure 2C**); a similar result was confirmed in the validating set (p = 0.029) (**Figure 2D**). In addition, the Kaplan–Meier curves of DFS of the two sets (p = 0.004 and 0.048, respectively) were in agreement with previous results (**Figures 2E, F**).

### Prognostic Value of the RBP-Based Classifier for Prediction of the Risk for Peritoneal Metastasis in Gastric Cancer

The 4-RBP-based signature, gender, and pathological stage were significantly related to peritoneal metastasis in the univariate logistics analysis. After the multivariate logistics regression analysis of the abovementioned factors, the 4-RBP-based signature and pathological stage were retained to be dependable factors for peritoneal metastasis in the training set. Except for gender, similar results were observed in the validating set (**Table 2**). Our result showed that the 4-RBP-based signature was an independent prognostic factor for peritoneal metastasis in gastric cancer in two sets. ROC curves were then plotted to appraise the competence of the 4-RBP-based signature to successfully predict the risk of PM in GC. The AUCs, in the training set and validating set (0.811 and 0.786, respectively), showed that the RBP-based classifier had similar predictive accuracy compared with the TNM staging (AUCs were 0.782 and 0.821 respectively) (**Figures 2G, H**). However, when we combined the RBP-based risk score and TNM staging to predict the risk of PM, the predictive capability was robustly enhanced. The AUC values of this built-up prediction model were 0.896 and 0.884 respectively in the training and validating set, suggesting better predictive accuracy. Whereafter, the 4-RBP-based risk score and TNM staging were used to structure a nomogram for predicting the risk of PM in GC patients (**Figure 2I**).

**Table 2:** Univariate and multivariate logistics analysis of the 4-RBP-based signature with peritoneal metastasis in the training set and the validating set.

Features	Univariate Logistics		Multivariate Logistics	
	HR (95% CI)	P	HR (95% CI)	P
**Traning set**				
Age (>65 vs. <=65)	1.786 (0.865, 3.688)	0.122	0.669 (0.230, 1.942)	0.459
Gender (Male vs. Female)	0.951 (0.915, 0.989)	**0.003**	0.989 (0.949, 1.030)	0.585
Pathological stage (I+II vs. III+IV)	3.743 (2.249, 6.231)	**<0.001**	4.169 (2.133, 8.148)	**<0.001**
4-RBP-based signature (High risk vs. Low risk)	51.419 (10.719, 246.657)	**<0.001**	37.604 (7.233, 195.503)	**<0.001**
**Validating set**				
Age (>65 vs. <=65)	1.139 (0.411, 3.156)	0.798	0.588 (0.138, 2.498)	0.472
Gender (Male vs. Female)	0.990 (0.952, 1.030)	0.554	0.992 (0.932, 1.055)	0.788
Pathological stage (I+II vs. III+IV)	4.855 (2.217, 10.633)	**<0.001**	4.658 (1.888, 11.488)	**<0.001**
4-RBP-based signature (High risk vs. Low risk)	16.119 (3.706, 70.105)	**<0.001**	14.411 (2.118, 98.044)	**0.006**

HR, hazard ratio; CI, confidence interval.

### Pathway Enrichment Analysis of Top 100 Correlated RBPs

**Figure 3:**
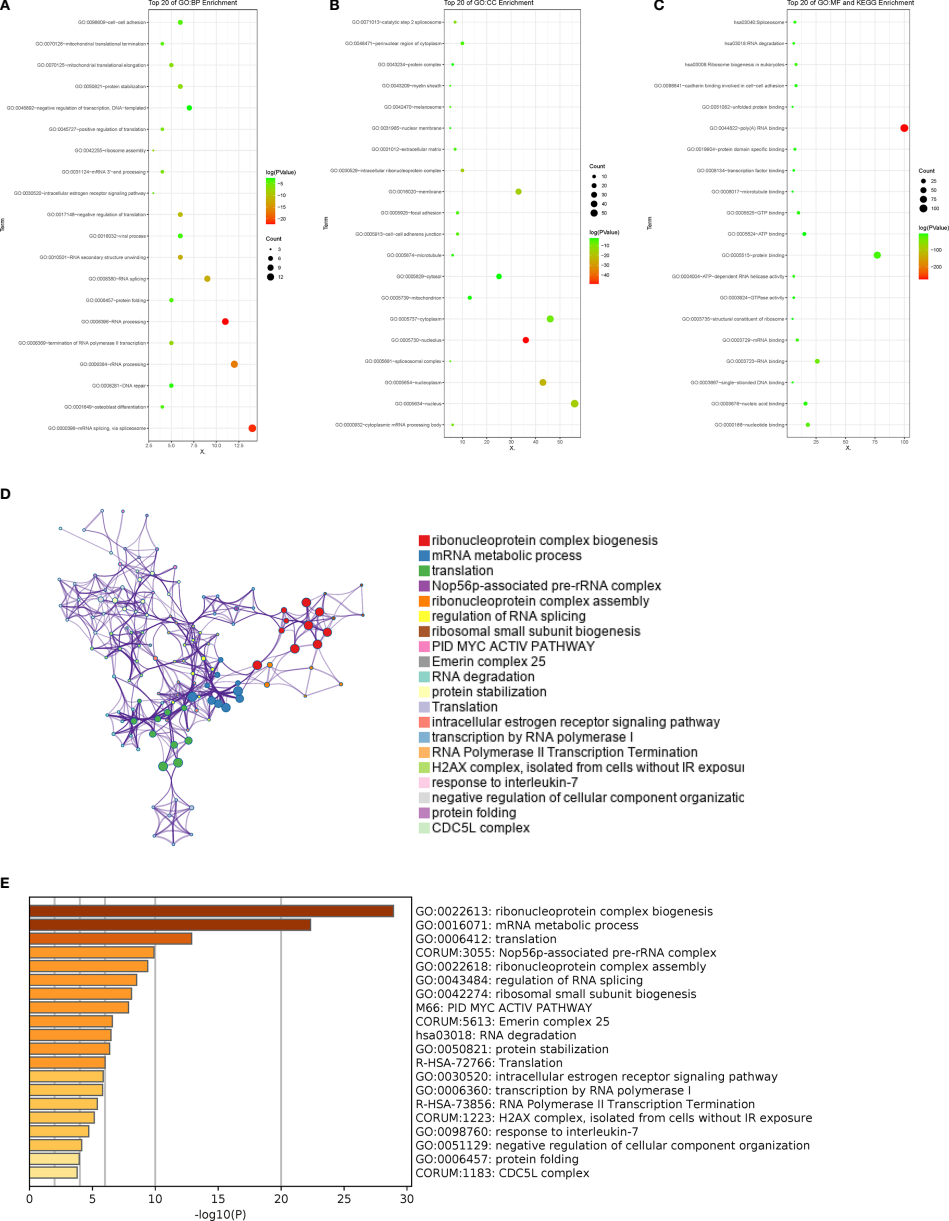
Pathway enrichment analysis of the related RBPs. **(A-C)** GO term and KEGG enrichment analysis performed by DAVID in BP, CC, MF and KEGG. **(D, E)** Pathways associated with the related RBPs were enriched by Metascape.

To explore the possible effect of the related genes on GC, DAVID and Metascape were used to perform function enrichment analysis. The results of DAVID revealed that the top 100 related genes were primarily enriched in mRNA splicing and RNA processing in biological processes (BP) (**Figure 3A**). In assessment of cell components (CC), the genes were mainly enriched in nucleolus and nucleoplasm (**Figure 3B**). While for molecular function (MF) and KEGG, the genes were generally enriched in poly(A) RNA binding (**Figure 3C**). The results of Metascape showed that the correlated genes mainly enriched in ribonucleoprotein complex biogenesis, mRNA metabolic process, translation, Nop56p-associated pre-rRNA complex, ribonucleoprotein complex assembly and so on, suggesting that these pathways were correlative with the PM of GC with (**Figures 3D, E**).

### Expression and Predictive Importance of the Four RBPs in Clinical Samples

**Figure 4:**
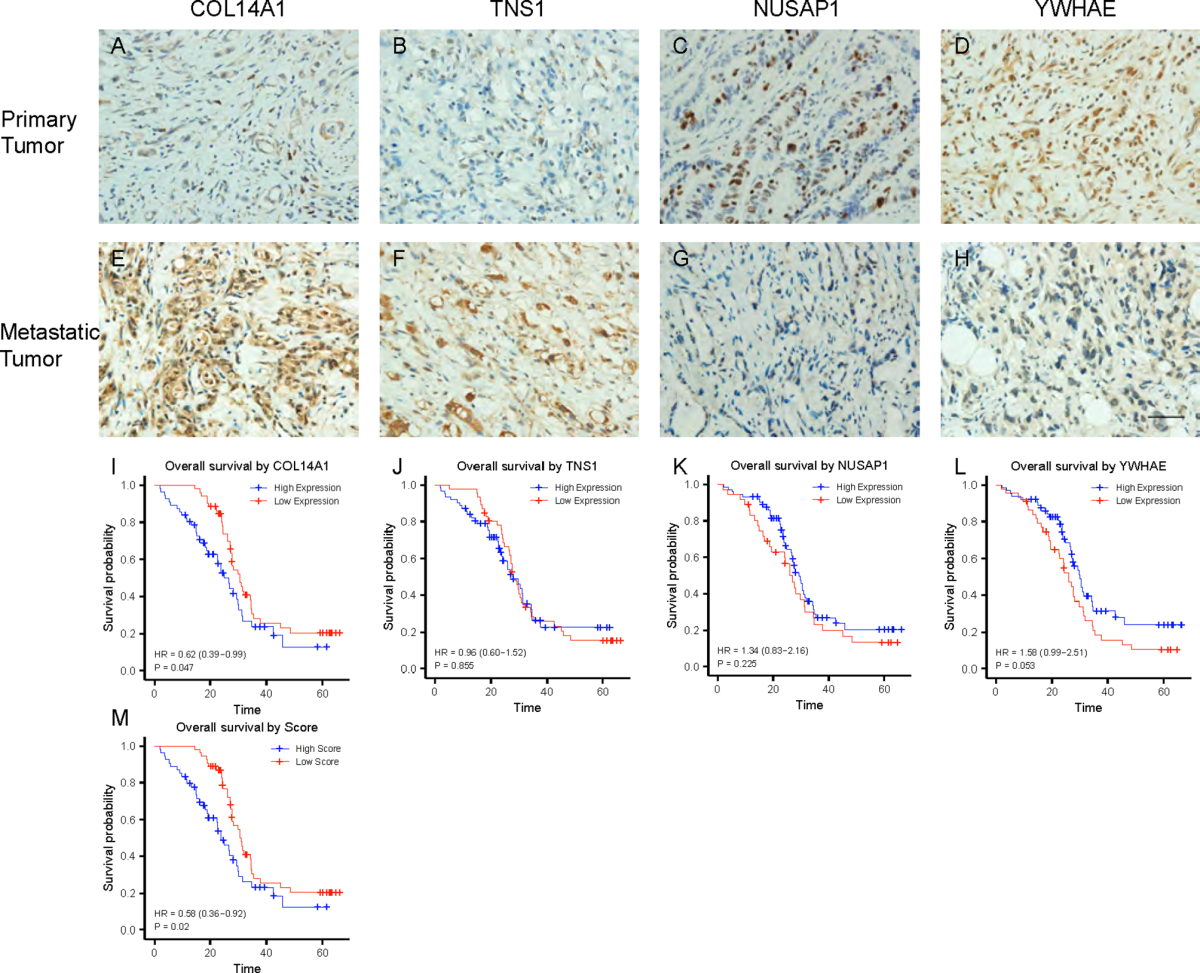
Expression and validation of the 4 RBPs in clinical samples. **(A–D)** IHC staining of COL14A1, TNS1, NUSAP1 and YWHAE in primary GC lesions. **(E–H)** IHC staining of COL14A1, TNS1, NUSAP1 and YWHAE in peritoneal metastasis lesions. Scale bar 1000μm. **(I–L)** Kaplan–Meier analysis for overall survival (OS) of GC patients based on different expression of COL14A1, TNS1, NUSAP1 and YWHAE. **(M)** Kaplan–Meier analysis for OS based on diverse scores.

We obtained 36 peritoneal metastatic samples of GC and 72 samples without PM. There was no significant difference between the two groups in gender, age, lymph node metastasis, HER-2, Ki67 (%), Lauren’s classification or lymphatic invasion assessed by IHC staining in 108 GC patients, 36 of which with PM (**Table 3**). However, we found that COL14A1 and TNS1 were over-expressed in peritoneal metastatic lesions compared with primary tumor tissues (**Figures 4A–D**); whereas conversely, NUSAP1 and YWHAE were over-expressed in primary tumor tissues compared with peritoneal metastatic lesions (**Figures 4E–H**), which is in accordance with our findings in the bioinformatic analysis above. Kaplan–Meier curves were separately drawn due to differential expression of the four RBPs in the GC patients (**Figures 4I–L**). It revealed that patients with a high expression of COL14A1 had shorter overall survival than those with low expression (p = 0.047), while there was no significance in the graph divided by the expression of TNS1, NUSAP1 and YWHAE (p = 0.855, 0.255 and 0.053, respectively). Nevertheless, according to the Kaplan–Meier curves, patients with high expression of NUSAP1 and YWHAE tended to have longer overall survival compared to those with low expression, which was consistent with our previous findings. Later, the patients were divided into high- score and low- score groups according to their 4-RBP-based signature. Kaplan–Meier curves further revealed that patients in the high-score group had shorter overall survival than those in the low-score group (p = 0.02) (**Figure 4M**).

**Table 3:** Clinical features of GC patients in the non-metastasis and peritoneal metastatic sets.

Features	Non-Metastasis (n=72)	Peritoneal Metastasis (n=36)	Pearson χ^2^	P
**Gender**				
Male	36	20	0.297	0.586
Female	36	16		
**Age**				
<=65	34	19	0.296	0.586
>65	38	17		
**Differentiation**				
Medium & High	28	17	0.686	0.408
Low	44	19		
**Lymphnode Metastasis**				
Positive	47	18	2.338	0.126
Negative	25	18		
**Lauren’s Classification**				
Intestinal	35	16	0.167	0.683
Diffuse & Mixed	37	20		

### Effects of the Four RBPs in Gastric Cancer *In Vitro*

**Figure 5:**
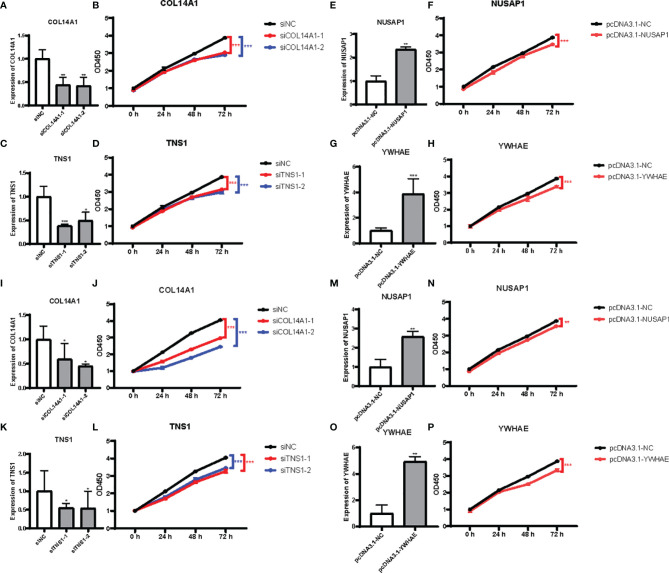
Effects of the 4 RBPs on GC cells *in vitro*. **(A–D)** Verification of knockdown of COL14A1 and TNS1, upregulation of NUSAP1 and YWHAE in AGS cell line. **(E–H)** Results of CCK-8 assays after transfection for 48 hours. **(I–P)** The same assays as aforementioned in MGC-803 cell line (N = 3). Data are presented as the mean ± SD values. *P versus < 0.05, **P versus < 0.01, ***P versus < 0.001.

To discover the function of COL14A1 and TNS1 in gastric cancer cells directly, we performed siRNA knockdown in human AGS cell line with two different siRNA sequences. Forty-eight hours after transfecting siRNA into the cancer cells, a drastic drop in the expression level of COL14A1 and TNS1 was assessed by qRT-PCR (**Figures 5A, C**). Then we measured cell proliferation by conducting CCK-8 assays. Results showed evident decrease of proliferation in AGS cells after knockdown of COL14A1 and TNS1 (**Figures 5B, D**). Subsequently, similar experiments of NUSAP1 and YWHAE were carried out with an overexpression plasmid, producing consistent outcomes (**Figures 5E–H**). Finally, repeated experiments were conducted in the MGC-803 cell line and accordant results were achieved (**Figures 5I–P**).

## Discussion

Mutations and alterations in RBP expression levels, which have been observed in many tumor tissues, are known to impact large sets of genes and contribute to tumor initiation and growth (17). Increasing literature has demonstrated that RBPs are of vital importance in the initiation, development, and recurrence of many tumors. For example, the RNA-binding protein NONO promotes breast cancer proliferation by post-transcriptional regulation of SKP2 and E2F8 (18). RBPs also play a vital role in the initiation of GC. It was reported that RBM5 downregulation was involved in GC progression, behaving as a tumor suppressor gene in GC (19). RBPs have the potential to be used as novel biomarkers as well. Musashi1 was reported to affect medulloblastoma growth *via* a network of cancer-related genes and was an indicator of poor prognosis (20). As a result, RBPs can regulate the biology of cancer and apparently possess potential as novel biomarkers.

Various models to predict the occurrence of GC have been created, including miRNA-based signatures, lncRNA-based monographs, even mixed-RNA-based classifiers (21–23). Each of them performs well at predicting the overall survival of GC. However, no RBP-based classifier for predicting the risk of PM in GC has been established yet. RBPs are a subset of molecules exhibiting different roles in regulating progression and development of malignancies. Taking the limited capability of a single RBP in prognostic prediction, we constructed a predictive model based on mRNA expression of four RBPs by univariate Cox regression and Lasso Logistics analysis. Patients were divided into two categories based on their median-risk score. After sample analysis, it turned out that high-risk patients have a greater possibility of PM than low-risk patients, suggesting that the signature had a robust ability for prediction of PM in GC patients. The 4-RBP-based score (AUCs being 81.1 and 78.6) presented a similar capability in forecasting PM risk as TNM staging (AUCs being 78.2 and 82.1) both in the training set and the validating set. Afterwards, we combined the 4-RBP-based signature together with TNM staging to evaluate prognosis on account of the extensive application of TNM staging in the clinic. It revealed that the combinative model (AUCs being 89.6 and 88.4) was more accurate than either the 4-RBP-based score or the TNM staging model employed separately. Therefore, we plotted a nomogram for practical application.

Four prognosis-related RBPs were selected to build the classifier, including COL14A1, TNS1, NUSAP1 and YWHAE. COL14A1 has been reported to exhibit a high mutation prevalence and an unexpectedly higher incidence of nonsynonymous mutations in GC, resulting in poor outcomes (24). There have also been studies demonstrating that downregulation of NUSAP1 suppresses cell proliferation, migration, and invasion *via* inhibition of the mTORC1 signaling pathway in gastric cancer (25). In addition, YWHAE silencing induces cell proliferation, invasion and migration through the upregulation of CDC25B and MYC in gastric cancer cells (26). However, functions of TNS1 in GC have not been explored yet. These articles provide evidence that support our model’s potential to assess the risk of PM in GC.

To verify the capacity of our 4-RBP-based signature, we brought 108 GC patients into our research, 36 of which were diagnosed with PM. IHC staining revealed that COL14A1 and TNS1 were over-expressed in peritoneal metastatic lesions relative to primary tumor tissues. On the contrary, NUSAP1 and YWHAE were over-expressed in primary tumor tissues compared to the peritoneal metastatic lesions, which was in accordance with our findings from bioinformatic analysis. Afterwards, the patients were divided into high- and low-score groups using the 4-RBP-based model. Kaplan–Meier curves revealed that patients in the high- score group had shorter overall survival than those in the low- score group (p = 0.011). Furthermore, we performed CCK-8 assays in AGS cells after knockdown of COL14A1, TNS1 and overexpression of NUSAP1, YWHAE. The results all showed decrease of proliferation.

In order to explore the biological function of the 4-RBP signature, we performed pathway enrichment analysis. Our results showed that those genes relevant to risk score were mainly enriched in cellular component organization or biogenesis, metabolic process, and positive or negative regulation of biological process, etc. Interestingly, we compared pathways predicted with annotations of these four RBPs in GeneCards6 and found that COL14A1 played an adhesive role by integrating collagen bundles, probably associated with the surface of interstitial collagen fibrils *via* COL1. Moreover, the COL2 domain may then serve as a rigid arm which sticks out from the fibril and protrudes a large N-terminal globular domain into the extracellular space, where it might interact with other matrix molecules or cell surface receptors. There was also a study characterizing the interaction of gastric cancer with peritoneal fibrosis which determined that TGF-b1 plays a key role in induction of peritoneal fibrosis, resulting from collagen formation and deposition, which in turn affected gastric cancer adhesion and metastasis *in vitro* and *in vivo (* 27). It was also reported that TNS1-silenced fibroblasts exhibited a strongly reduced capacity to contract collagen gels (28), probably leading to some effects on PM of GC. Furthermore, it has been reported not long ago that YWHAE silencing induced cell proliferation, invasion, and migration through the upregulation of CDC25B and MYC in gastric cancer cells in accordance with our conclusion above. However, NUSAP1 was demonstrated to facilitate cell proliferation, migration, and invasion *via* inhibition of the mTORC1 signaling pathway in gastric cancer, which was contrary to our results (25). So far, we have found no reasonable explanation for the exact mechanisms of these RBPs, indicating that more research is required to investigate their specific roles in PM of GC.

## Conclusion

In general, we identified four RBPs associated with risk of PM of GC. We then constructed a 4-RBP-based classifier to help predict the prognosis, ultimately providing a tremendous help in clinical decisions. Our results showed that this classifier can successfully categorize patients into high-risk and low-risk groups with large differences and promote the predictive ability of the current TNM staging system. Nevertheless, large-scale, multi-center, and prospective studies are necessary to confirm our results before the 4-RBP-based signature is applied in the clinic.

## Data Availability Statement

The datasets presented in this study can be found in online repositories. The names of the repository/repositories and accession number(s) can be found in the article/**Supplementary Material**.

## Ethics Statement

The studies involving human participants were reviewed and approved by the Ethics Committee of Renmin Hospital of Wuhan University. Written informed consent for participation was not required for this study in accordance with the national legislation and the institutional requirements.

## Author Contributions

YJ was responsible for designing the protocol, writing the protocol and report, conducting the search, screening potentially eligible studies, extracting and analyzing data, interpreting results, updating reference lists, and creating the tables. FC was responsible for designing the protocol and screening potentially eligible studies. She contributed to writing the report, extracting and analyzing data, interpreting results. XR contributed to data extraction and provided feedback on the report. YY, JL, and JWY contributed to arbitrating potentially eligible studies, extracting and analyzing data and interpreting results. JPY and QT provided feedback on the report. All authors contributed to the article and approved the submitted version.

## Funding

This work was supported by the National Natural Science Foundation of China (No. 81172186) (QT), by the Natural Science Foundation of Hubei Province (No. 2018CFB504) (QT), and by the Guidance Foundation of Renmin Hospital of Wuhan University (No. RMYD2018M67) (QT).

## Conflict of Interest

The authors declare that the research was conducted in the absence of any commercial or financial relationships that could be construed as a potential conflict of interest.

## Publisher’s Note

All claims expressed in this article are solely those of the authors and do not necessarily represent those of their affiliated organizations, or those of the publisher, the editors and the reviewers. Any product that may be evaluated in this article, or claim that may be made by its manufacturer, is not guaranteed or endorsed by the publisher.
